# A feasibility study to evaluate a purposeful walk intervention with a distance goal using a commercially available activity monitor in elderly people post total hip replacement surgery

**DOI:** 10.1177/20556683231195927

**Published:** 2023-08-22

**Authors:** Shayan Bahadori, Jonathan Mark Williams, Sarah Collard, Ian Swain

**Affiliations:** 1 Orthopaedic Research Institute, 6657 Bournemouth University, Bournemouth, UK; 2 Faculty of Health and Social Sciences, 170790 Bournemouth University, Bournemouth, UK; 3 Faculty of Science and Technology, 276175 Bournemouth University, Poole, UK

**Keywords:** Total hip replacement, activity monitor, walking activity, gait analysis

## Abstract

**Introduction:**

Total hip replacement (THR) is performed in an increasing number of individuals around the world and while improvements in pain reduction and long-term enhancement of muscle strength are well documented, the improvement in daily activity does not follow the same trend. This study aimed to determine the feasibility of a 5-week intervention where a personalised outdoor walking distance is monitored using a commercial activity monitor (Fitbit Charge 4).

**Method:**

Data was collected on gait and activities of daily living using patient reported outcome measures. Following the completion of the intervention period, participants took part in a semi-structured interview to voice their opinion on the use of the activity monitor, their experiences, and any challenges in order to assess the feasibility of the intervention. All quantitative data were presented descriptively, using appropriate summary statistics. Interviews were analysed using thematic analysis.

**Results:**

Five participants who had undergone total hip replacement surgery within the postoperative period of 3 to 6 months were recruited from the local community.

**Conclusion:**

The findings suggest that the intervention was feasible and that it encouraged all participants to increase their daily activity. Therefore, it can be concluded that a follow-up effectiveness trial is warranted.

## Introduction

Total hip replacement (THR) is performed in an increasing number of individuals around the world with the primary aim of reducing pain and improving function.^1^ The National Joint Registry (NJR)^2^ reported that over the last 3 years, 250,278 total hip replacement procedures were performed in the UK on individuals with a median age of 69, and this figure is predicted to rise by 208% by the year 2035. Meanwhile, with the cost of the operation being around £7500,^3^ combined with the time taken to return to normal activities and work, THR places a significant financial burden on the National Health Service.

While improvements in pain reduction, range of motion of hip joints, and long-term improvement of muscle strength are well documented,^4–6^ the improvement in gait and in particular the walking ability does not follow the same trend.^7^ A recent study monitored the first 3 months of the recovery post THR and data showed that the number of steps after THR decreases temporarily after surgery and does not reach pre-surgery levels even at 3-month post-surgery.^8^ Other studies looked at a longer period and found that this deficit even remains at 1-year post-surgery^9–11^ and also few meet the physical activity guidelines recommended by the World Health Organization (WHO).^12^

There are currently no recommendations for the optimal amount of walking that should be recommended after THR surgery. A recent report^13^ including both groups of THR patients (before and after surgery) and healthcare professionals (physiotherapists and surgeons) concluded that walking freely i.e. long outdoor walks without pain, is one of the main reasons that people undergo THR surgery, and therefore should be recognised and monitored as a factor to a positive long-term outcome measure. Furthermore, another study^14^ reported that an ability to walk even a short distance outdoors can be meaningful for successful and independent living at home among the THR group, as well as enhancing their physical function.^15^

The availability of commercially available wearable devices, such as activity monitors, allows objective monitoring of daily activities such as walking. In addition to their growing popularity,^16^ these devices are equipped with a wide variety of different sensors such as the Global Positioning System (GPS), and algorithms to collect and display physical activity data in indoor and outdoor settings. In an earlier study,^17^ the precision, accuracy, and consistency of different activity monitors, including the Fitbit Charge 4 (FC4) device was explored in a variety of settings and walking speeds. The findings from this study suggested that the FC4 is the most suitable activity monitor for a study involving the elderly population, and with the best accuracy and precision in measuring the distance walked using the GPS sensor. Whilst, research has provided evidence to support the use of wearable activity monitors in maintaining good health in older adults,^18,19^ when it comes to THR studies,^20,21^ there is limited evidence to support its benefits. Furthermore, for all of these studies, the focus has been merely on step count and has not addressed the main gait adaptations e.g. shortened stride length,^7,22,23^ which persist long term after surgery. The benefits of distance-based walking in contrast to time or step count has already shown benefits in reducing cardiovascular disease,^24^ improving stride length in older adults^25,26^ as well as increasing the walking efficiency pre and post-THR surgery.^27^ Therefore the concept of monitoring the distance walked in an outdoor setting, using the GPS sensors of a commercially available activity monitors, emerges as a potentially motivating factor. However, before implementing new methods to promote outdoor walking, it is important to gain understating of the feasibility (uncertainties around recruitment, outcome measure, adherence, and acceptability etc.) of such a proposition.

This study aimed to determine (1) the feasibility of an intervention where walking distance is used as a parameter to increase daily walking activity using a commercially available activity monitor (FC4) in THR patients 3–6 months post-surgery, (2) explore the barriers and facilitators to implement the intervention, and (3) assess the feasibility of the recruitment and the adherence to the use of the FC4 activity monitor, and appropriateness of different outcome measures. Throughout this paper, we will refer to the outdoor walk that is recorded with a GPS sensor as a ‘purposeful walk’.

## Methods

### Study design

This was an investigator-initiated, single-center feasibility trial with full ethical approval granted by the Bournemouth University Research Ethics Committee (ref: 42236) and prepared in accordance with STROBE guidelines for reporting feasibility studies.^28,29^

### Participants

Table 1 provides full eligibility criteria for the participants in the study. Participants were all recruited through publicising tools such as Twitter posts, and posters shared on the University channels (Bournemouth University research blogs, the Public Involvement in Education and Research (PIER) group), University of Third Age, and communities of older adults (e.g., local indoor bowling clubs). Those interested in the study were asked to contact the lead researcher (SB) for more information. Once an individual had expressed an interest in taking part, the lead researcher emailed the individual a copy of the participant information sheet. To comply with Good Clinical Practice (GCP) guidelines, the participant was given 48 h to consider their participation in the study. The lead researcher then contacted the participant to undertake initial eligibility screening and to attend a baseline assessment.

**Table 1.** Eligibility criteria.

Inclusion Criteria	• Male and female, aged 60 years and over;
	• 3 to 6 months post unilateral total hip replacement surgery for osteoarthritis;
	• Can provide verbal confirmation that they have been discharged from their surgical care;
	• Capable of independent walking;
	• Capable of completing the activity diary independently;
	• Have access to a smartphone or computer;
	• Willing to complete the trial protocol.
Exclusion Criteria	• Unable to provide informed consent;
	• Unable to complete follow-up (insufficient English, lives overseas, unable to return easily);
	• Not physically able to use Grail gait lab;
	• Systematic disease affecting walking ability (chronic obstructive pulmonary disease (COPD), congestive heart failure (CHF), chronic kidney disease (CKD), Parkinson’s Disease, cerebral palsy, multiple sclerosis etc.);
	• Requiring revision hip replacement;
	• Previous hip replacement (resurfacing or THR) on the contralateral side;
	• Known metastatic tumour involving the hip.

### Sample size

Five participants were chosen to take part in this feasibility study. Given this was a feasibility trial, a convenience sample size was selected, and a formal calculation was not carried out.

### Setting

The study was carried out at the Orthopaedic Research Institute at Bournemouth University. Following taking informed consent, data were collected on gait, and on activities of daily living using patient reported outcome measures (PROMS) questionnaires. Participants were invited to attend a final assessment at 5 weeks from their baseline appointment where their baseline measures were repeated. In addition, participants were asked to keep a diary of their daily walking activities and the intensity of their walk. After the intervention period was complete, participants were invited to attend an interview with the lead researcher in which they were able to openly express their thoughts on the use of the activity monitor, their compliance, practicality, and the usefulness of the intervention.

### Objective measurement tool

The study period and visit schedules are summarised in Table 2. The choice of key outcome measures was sought by a search conducted on The COMET database (Core Outcome Measures in Effectiveness Trials; www.comet-initiative.org). However, no results were found in regard to the studies including THR participants.

**Table 2.** Visit schedule.

	Study period							
	Enrolment	Allocation	Study intervention					Post-intervention
TIMEPOINT	*Call 1*	*Visit 1 Baseline*	*Week 1*	*Week 2*	*Week 3*	*Week 4*	*Week 5*	*Visit 2 - Follow up assessment*
*Eligibility screen*	X	X						
*Informed consent*		X						
*Enrolment*		X						
INTERVENTION								
*Purposeful intervention*			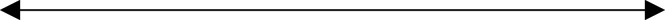					
ASSESSMENTS								
*Gait analysis*		X						X
*HOOS*		X						X
*PASE*		X						X
*mGES*		X						X
*Activity Diary*			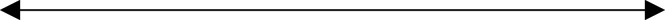					X
*Fitbit Charge 4*								X
*Interview*								X

#### Activity monitor, FC4

FC4 was identified as the most suitable and precise activity monitor for a study involving the elderly population, in measuring the distance walked using the GPS sensor. Adherence was assessed in terms of the usage and repeated usage of the FC4. This data were downloaded by the lead researcher at the end of each day using the Fitbit app which has been connected to the study’s Fitbit account.

#### Gait analysis

The Gait Real-time Analysis Interactive Laboratory (GRAIL, Motekforce Link, Amsterdam, the Netherlands) system was used to carry out the gait analysis. GRAIL combines a fully instrumented treadmill with a self-paced option, as described by Sloot et al.^30^ The treadmill is feedback-controlled, which allows participants to walk at their preferred speed. It compromises a virtual environment, 10-camera Vicon MX optical infrared tracking system (Oxford Metrics, UK), and a split-belt instrumented treadmill. The gait analysis was carried out as per the protocol published on gait analysis using the GRAIL system.^31^ However, only Spatio-temporal data (walking speed, cadence and step length of the operated side) which are directly related to the walking pattern of participants were recorded for analysis. Participants were asked to wear comfortable shoes and tight clothing (such as cycling shorts or leggings). They were fitted with 25 passive reflective markers using the Human Body Model (HBM) lower body marker set.^32^ Following an acclimatisation period, three sets of 25 gait cycles were recorded.^31^ The reliability of the GRAIL system in self-paced mode walking speed^33^ has been previously reported and it is recommended that a minimum of 23 gait cycles should be captured to attain the characteristics of individuals’ walk.^34^ Spatial-temporal gait parameters for all participants were exported as a. CSV file and analysed in Matlab R2019b (The Mathworks Inc., USA). Gait analysis was undertaken as it has proved a valuable tool in identifying objective data on individual walking patterns and modalities before and after THR.^35^

### Patient reported outcome measures

PROMs were selected to give a broad understanding of the level of daily activity, functional limitation, occupational activity, and level of confidence in walking 3 to 6 months post-THR surgery.

#### Hip-related disability

Hip-related disability was assessed using the Hip Disability and Osteoarthritis Outcome Score (HOOS) questionnaire^36^ (Appendix A). The tool is validated in a sample of participants after THR surgery^37^ and was perceived as relevant and is intended to be used to assess the individual’s opinion about their hip and associated problems, and to evaluate symptoms and functional limitations related to the hip during a therapeutic process. The HOOS includes 40 items with five possible responses, graded from 0 to 4 (0 points = worst possible score; 100 points = best possible score). To answer the questions, standardized answer options are given in 5 Likert-boxes with scores from 0 to 4 (no, mild, moderate, severe and extreme).To provide meaningful information to support the clinical effect of the 5-week programme on individuals, the minimal clinical important difference (MCID) for the HOOS was considered to be 24.^38^

#### Physical activity levels

Activity levels were measured using the Physical Activity Scale for the Elderly (PASE) questionnaire^39^ (Appendix B). The self-administered questionnaire is a valid and reliable tool for adults with hip osteoarthritis, that consists of 12 questions regarding the duration, frequency, exertion level, and amount of physical activity undertaken during a 7-day period.^40^ It was perceived relevant as it was designed to assess a broad range of activities, including household tasks, occupational activities, active transport, and sports and exercise in older adults, and therefore given our inclusion criteria it provides an insight into such age range who undergone THR surgery. It uses frequency, duration, and intensity level of activity over the previous week to assign a score, ranging from 0 to 791, with a higher score indicating greater physical activity.^39^

#### Gait efficacy

The modified Gait Efficacy Scale (mGES)^41^ is a 10-item measure that addresses older adults’ perception of their level of confidence in walking during challenging circumstances. The items include walking on a level surface and on grass, stepping over an obstacle, stepping up and down a curb, ascending and descending stairs (with and without a handrail), and walking over a long distance (Appendix C). The items are scored individually on a 10-point Likert scale, with 1 denoting no confidence and 10 representing complete confidence, giving a total score range of 10 to 100, with 100 representing complete confidence in all tasks.^41^ This questionnaire was particularly relevant as it provided a subjective insight into participants walking capabilities to compliment the gait analysis objective evaluation. The mGES is validated in studies including older adults,^42^ total knee replacement patients,^43^ and individuals undergoing lower limb fixation surgery^44^ and is perceived feasible in other orthopaedic related studies such as THR.

### Activity diary

Participants were given an activity diary to record their daily walking activity (Appendix D). They were asked to record the amount of distance walked in kilometers (km) as reported on their activity monitor after each purposeful walk. They were also asked to rate the intensity of their walking using the Borg scale^45^ following each purposeful walking session. To further explore the barriers and facilitators to implement the intervention, the activity diary also had sections where participants were able to document their feelings/conditions which may have affected their attempts to do their daily purposeful walk. Participants brought their activity diary to the interview in order to remind themselves of any challenges or positive experineces they faced during the 5 week intervention.

### Interviews

Following the intervention completion, in order to qualitatively explore the feasibility of the intervention, all participants were invited for a semi-structured interview held at Bournemouth University. The use of a semi-structure interview is proven to be an effective method to (1) collect qualitative, open-ended data; (2) explore participant thoughts, feelings, and beliefs about a particular topic; and (3) delve deeply into participant’s challenges and experiences.^46^ A topic guide (see Appendix E) was designed to inform the study design of any future trial by determining which elements of the intervention worked well for participants, and which needed adjustment or further development. Participant feedback was analysed using thematic analysis.^47,48^ The six phases of the thematic analysi,^48^ (1) familiarisation with the data, (2) generating codes, (3) searching for themes, (4) reviewing themes, (5) defining, and (6) naming themes, were followed. The recording was anonymised and transcribed discussions were read through several times by the lead researcher to become familiar with the data and were organised using Microsoft Excel Version 2108. Codes were thereafter created, and similar codes were organised into potential themes. The sessions took around 25 to 35 min and were conducted in June 2022.

### Purposeful walking intervention

**Figure 1.**
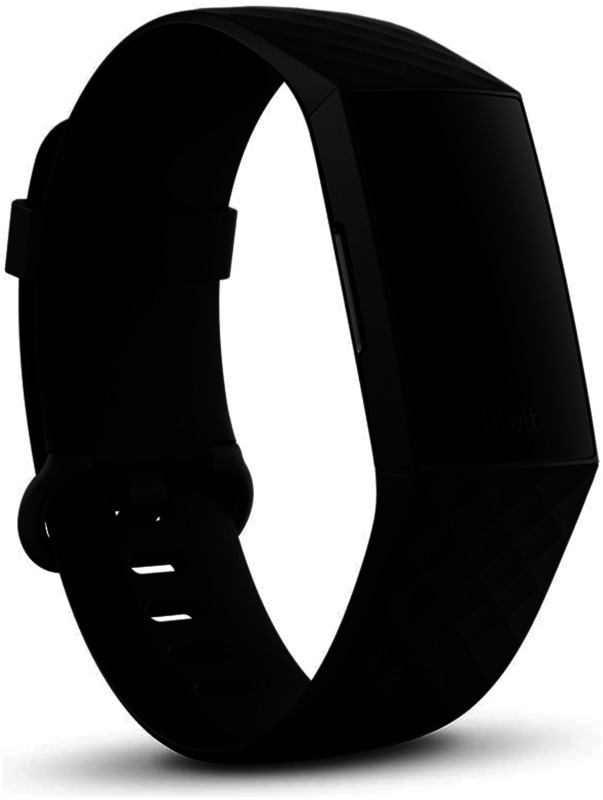
Fitbit charge 4 (FC4).

The purposeful walking intervention in this study was monitored using the FC4 activity monitor (Figure 1). Participants wore the FC4 activity monitor for 5 weeks in total. In the first week, participants wore their FC4 activity monitor in order to understand the participant’s post-surgical walking distances. In week two, a target distance was calculated to increase the weekly walking distance by 10% and was divided by seven to calculate a daily distance for that week. In the weeks thereafter, if participants met their target, a new purposeful distance target was calculated to increase the participant’s walking distance by a factor of 10% from the previous target. If the participant did not meet their target, the daily distance goal they were assigned the previous week remained in place. Participants were contacted through the FC4 Fitbit app on a weekly basis throughout the study and were given their daily goals for the upcoming week. The FC4 activity monitor was worn on the wrist of the non-dominant hand continuously during the study period. Participants were shown how to charge and operate the FC4 activity monitor and were given a copy of a simple instruction manual to take with them.

### Descriptive analysis

All data were analysed using Microsoft Excel Version 2108 (Microsoft Corporation, 2022, Retrieved from https://office.microsoft.com/excel). As this is a feasibility study, all quantitative data (gait, and PROMs scores) was presented descriptively, using appropriate summary statistics. In the absence of any direct guidance associated with the development of walking post THR surgery over a period of weeks, the feasibility for using a purposeful walk with target distances in individuals post THR was determined if individuals managed to increase their baseline purposeful walk by more than 40% (4 weeks multiply by 10%) from their baseline. Adherence to the intervention was assessed in terms of the daily purposeful walk amount that was recorded using FC4 and reported through the Fitbit App. Full adherence was achieved if all participants reported their daily purposeful walk amount, and no data were missed. Recruitment was assessed based on the time needed to recruit the study participants, with 1 participant per week being an acceptable recruitment rate.^49^ The feasibility of different outcome measures was assessed through appropriations of collected data, and the practicality of delivering the assessments such as the time it took for each assessment.

## Results

### Recruitment

Thirteen participants contacted the lead researcher over a period of 59 days of which, eight did not meet the inclusion criteria. Reasons for exclusion were: four did not have a smartphone, one suffered from systematic disease, one did not speak English, and two were under the age of 60 years old.

### Participant demographics

Five adults (2 Male, 3 Females, average age 68 ± 5.7 years old, average BMI 27.8 ± 7.2 kg/m^2^) were recruited to take part in this study. Table 3 summarises the participant's demographic information.

**Table 3.** Participants’ demographics information.

Participant	Age	Months Post Op	Height (cm)	Weight (kg)	BMI (kg/m^2^)	Gender
1	73	5	178.5	88.0	27.6	Male
2	74	4	163.4	58.0	21.7	Female
3	66	3	164.5	64.6	23.9	Female
4	60	4	178.4	127.6	40.1	Male
5	67	5	164.1	68.8	25.5	Female

### Feasibility and adherence of the intervention

**Figure 2.**
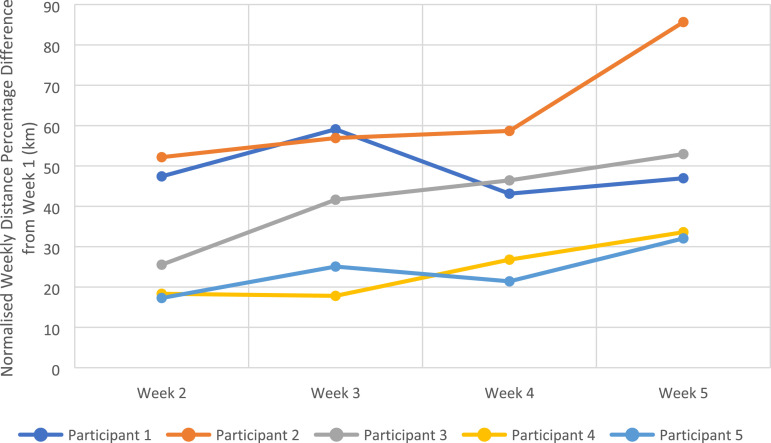
Normalised percentage difference of purposeful walking distance achieved by each participant per week.

Figure 2 outlines individuals’ percentage difference of the weekly total purposeful walk normalised to their baseline week (week 1). Results suggest a weekly increase of 10% to individuals’ baseline walking distance was achieved, with all participants adherent to the use of FC4 and reporting a maximum purposeful walking distance of more than 40% from their baseline amount. Except Participant 1 who achieved his maximum purposeful walk at week 3 (70% increase from baseline), all participants achieved their maximum walking amount at week 5.

### Feasibility and practicality of different outcome measures

The feasibility of the various outcome measures was assessed through the appropriateness of collected data, and the practicality of delivering the assessments. On average it took approximately 45 min for the baseline and follow-up assessment sessions.

#### Activity monitor

The purposeful walking intervention in this study was monitored using the FC4 activity monitor. Throughout the study, participants were able to use the FC4 easily, record all of their purposeful walks, and report the distance of their daily purposeful walks. Thus, it can be concluded that as an activity monitor, the selection of FC4 with the target population is appropriate.

#### Gait analysis

**Figure 3.**
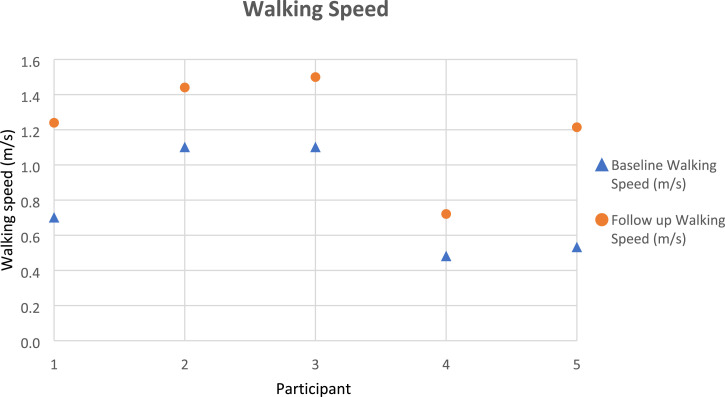
Walking speed gait data for each participant.

**Figure 4.**
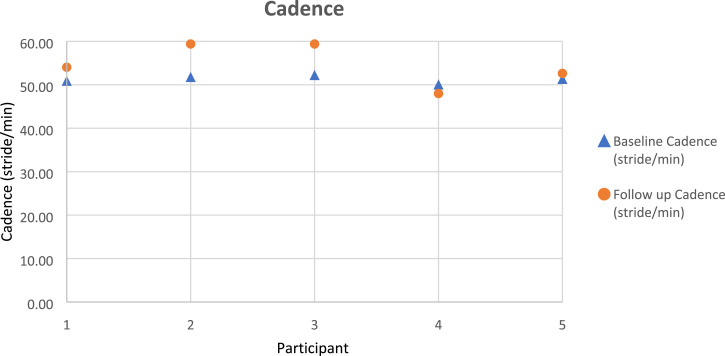
Cadence gait data for each participant.

**Figure 5.**
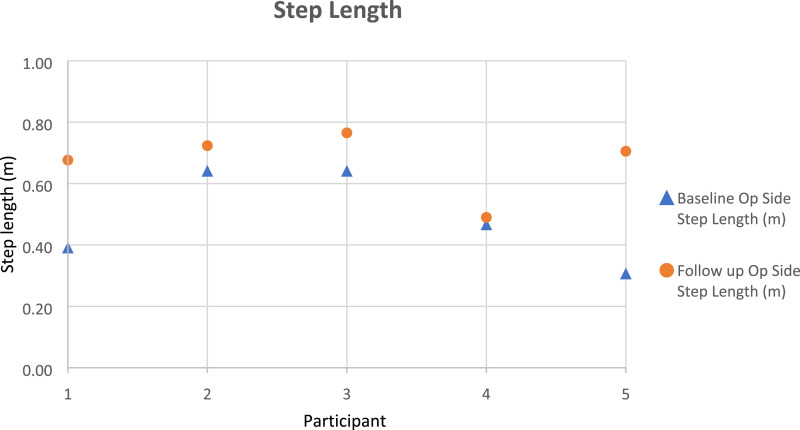
Step length of the operated side gait data for each participant.

Gait parameters (walking speed, cadence, step length) were recorded as per the protocol, using the GRAIL system, and took approximately 20 min to complete. Figures 3–5 outline each participant’s gait changes from pre to post-intervention. These findings suggest improvement of step length (operated side), walking speed, in all participants and cadence in four out of five participants following the purposeful walk intervention.

#### Patient reported outcome measures

**Figure 6.**
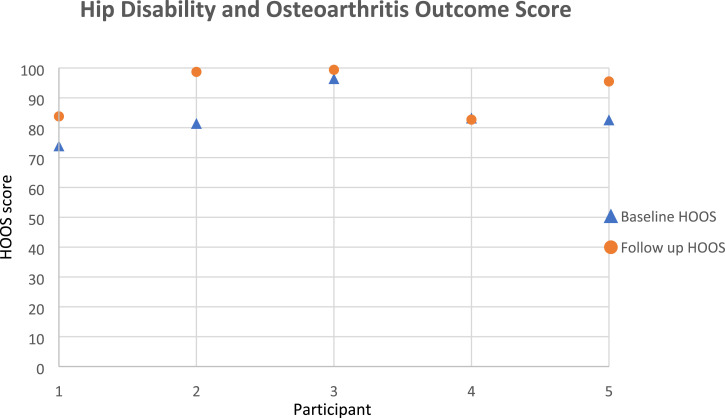
Hip disability and osteoarthritis outcome score (HOOS) data for each participant.

**Figure 7.**
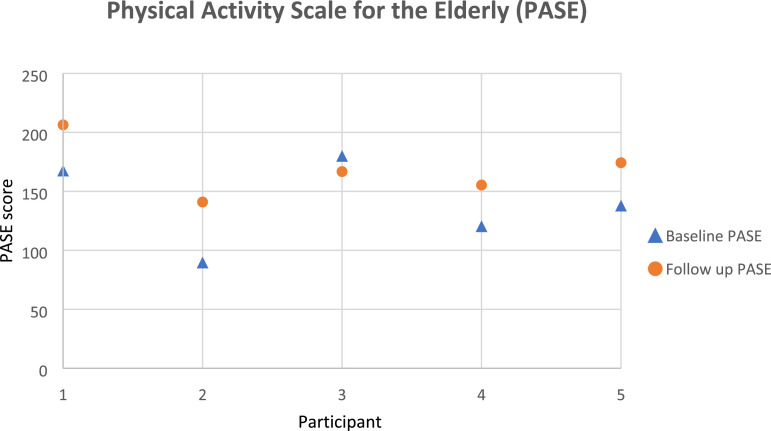
Physical activity scale for the elderly (PASE) data for each participant.

**Figure 8.**
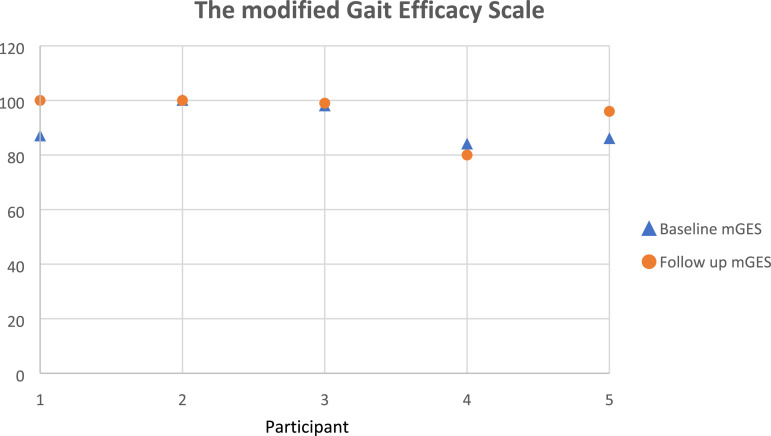
The modified gait efficacy scale (mGES) data for each participant.

Figures 6–8 show participants’ data for HOOS, PASE, and mGES respectively. All participants were able to complete all of the questionnaires as per the protocol. Except for the PASE score for participant 3, and the mGES score for participant 4, PROMs data indicated an improvement in all participants. The MCID for pre to post-intervention was not seen in the HOOS score in any of the participants.

### Qualitative findings

#### Activity monitor

**Figure 9.**
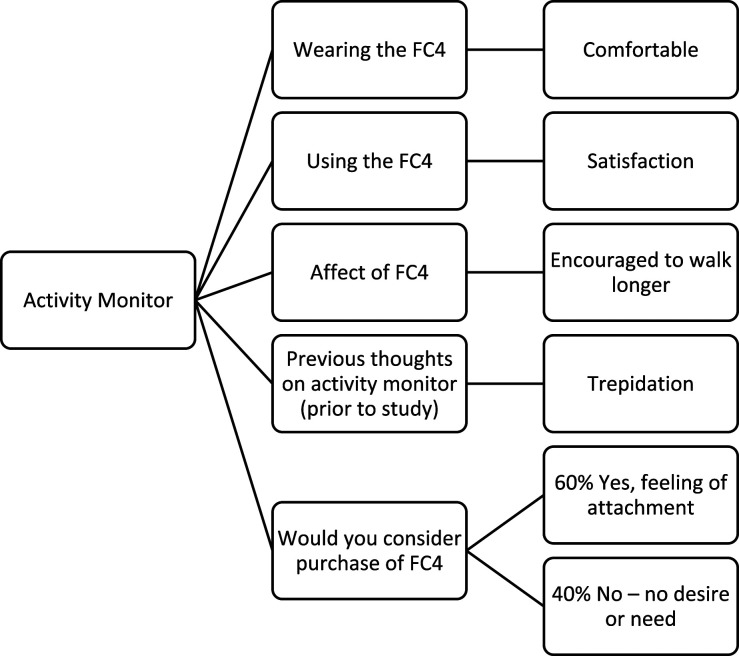
The codes and themes related to the subject discussion, activity monitor.

The codes and themes relating to the activity monitor, are illustrated in Figure 9. Participants expressed that the FC4 was comfortable to wear on the wrist and encouraged them to walk further and increase their daily physical activity. The theme for the overall use of the FC4 was *satisfaction*. However, there were suggestions concerning the difficulties around the GPS signaling (Participants 1, and 3). Subsequently, there were positive comments in relation to the use of GPS and pleasure in accessing the daily map of the purposeful walk (Participant 2). Additionally, being able to show others how much distance they had walked during the day was highly valued by participants. It provided evidence and reason for their need for rest, regardless of whether they needed to put their feet up after a day at work or to stop walking after an entire day of sightseeing while on vacation. Previous experience regarding *the use of such activity monitors* was also discussed and participants mentioned “*trepidation”* (Participant 5) feelings in relation to this matter. However, post participation in the study, they all enjoyed using the FC4 and would consider the future purchase of such activity monitors. The codes and themes related to the activity monitor, are illustrated in Figure 9. Quotes from participants concerning the activity monitor are outlined below.

*“I would not say it encouraged us to walk (wife & I ) as we usually have a daily walk. I would say - however it encouraged us to have a longer walk - & we tried our best to meet my set target for that week.”*

*Participant 1*

*“Yes I think I would (consider buying one). I will miss wearing it and seeing the maps of my walks.”*

*Participant 2*

*“The activity monitor did encourage me to walk and achieve my daily goals. It is an excellent piece of kit and I enjoyed the email feedback when my goals had been achieved. I was very impressed with the activity monitor.”*

*Participant 3*

*“I hadn’t given them any thought before the study at all. I intend to get one after I have given yours back to you.”*

*Participant 4*

*“No, I don’t think I would buy one. But it proved a point about exercising to return to fitness, as it renewed my cognisance of the benefits of moving more, and sitting down less.”*

*Participants 5*

#### Purposeful walking intervention

**Figure 10.**
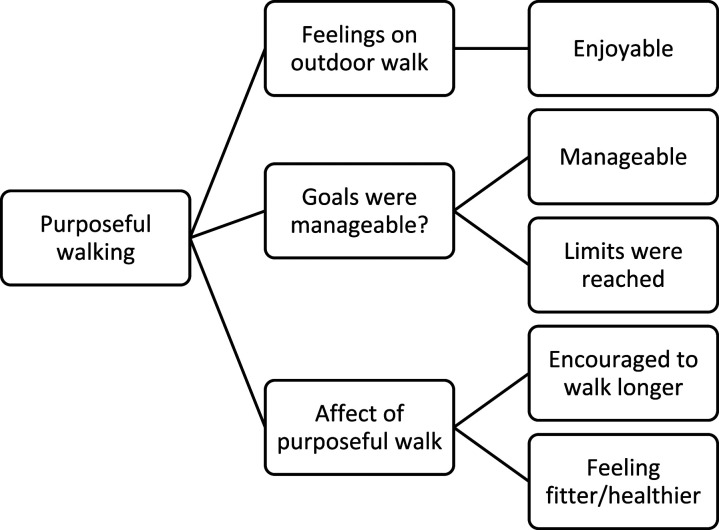
The codes and themes related to the subject discussion, purposeful walk.

Participants felt enthusiastic, excited, and enjoyed the purposeful outdoor daily walks. Personalised daily distance targets were manageable for most of the participants and the weekly gradual increase allowed the participant to push beyond their self-believed limit. Allowing time and having the purposeful walk planned into the daily schedule was also deemed feasible by all except one (Participant 1). This participant’s (Participant (1) target was 9.4 km on week three and they found it challenging to fit it into the daily schedule. Conversely, another participant (Participant (2) reached 9.4 km on week five and she was able to achieve this daily distance and expressed her joy in doing so. The purposeful walk allowed participants to feel they had regained the muscles that they had lost post-surgery, as well as feeling fitter physically and mentally by being connected with outdoor nature again. The codes and themes related to the purposeful walking intervention, are illustrated in Figure 10. Quotes from participants concerning the purposeful walking intervention are outlined below.

*“The beginning (4 km) was easily manageable but 9 km a day a long time to fit into my daily schedule”*

*Participant 1*

*“I really enjoyed going out for my outdoor walks and I really enjoyed my early morning walks as it was so therapeutic to listen to the birds singing early morning. Part of my study was carried out on a cruise ship, to be at sea and completing an outside walk is quite magical, listening to the waves lapping the water and the sun shining off a clear blue sea, combined with a gentle breeze was very exhilarating.”*

*Participant 2*

*“The daily distance goals were extremely helpful in increasing my daily activity. It made me feel fitter, encouraged me to walk further daily and I feel healthier in myself plus I feel more toned up. It has encouraged me to walk on a daily basis.”*

*Participant 3*

*“Early ones (daily distance goals) were very manageable. The 4.4* *km per day I think at the moment is my limit for a while, easily achieved at work not so out of work but I’m stubborn and I had to finish my given goal.”*

*Participant 4*

*“Yes, they were helpful (daily distance goals). As my leg muscles strengthened, my hip gave me less pain, so I was able to look at my goal as a challenge to aim for and surpass, and grow more confident in realising that I still had the ability to move nearly as well as before the hip replacement. I felt safe in the knowledge that I would not be asked to do something that was too much for me to achieve - after the operation, I was unsure if I would damage my new joint if I did too much, or too little moving around.”*

*Participant 5*

#### Outcome measures

**Figure 11.**
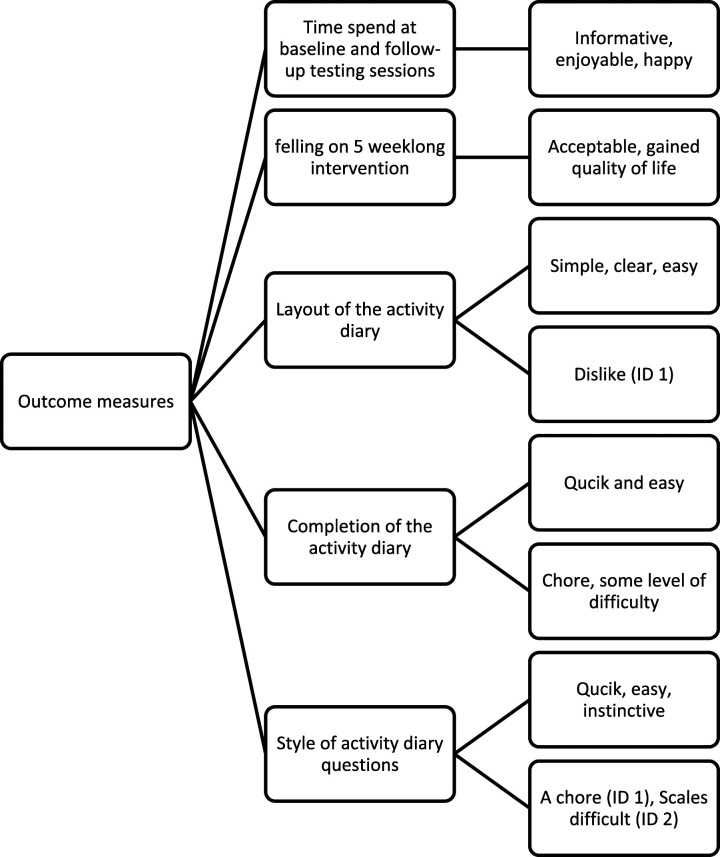
The codes and themes related to the subject discussion, outcome measures.

Participants were interviewed about their feeling on the time spent during the testing sessions, the duration of the intervention (5 weeks), the layout of the activity diary, their feelings on completing it as well as the styles of its questions. All participants were happy with the duration of the baseline and follow-up testing sessions at the Institute. Participants also were happy with the duration of the whole intervention and felt it passed by very quickly. They felt 5 weeks was a small amount of time commitment in comparison to the benefit they gained in taking part in this study. However, they found the Likert scale of the activity diary irrelevant and difficult to complete and preferred the section in which they can openly write any condition or feeling which may affect their daily outdoor walk. Overall, there was a mixture of feelings about the layout of the activity diary. One of the participants (Participants 1) did not enjoy paperwork and therefore found the activity a chore. Participant 2 also provided feedback on the layout design and suggested leaving more spaces in the diary table where they can express their daily feeling on conditions affecting their outdoor walk. All others expressed that the layout was simple, questions were clear, and completion was easy. The codes and themes related to the Outcome measures are illustrated in Figure 11. Quotes from participants concerning the Outcome measures are outlined below.

*“I am afraid I don’t do very well with paperwork & although keen at first - need to report my attitude has not changed.”*

*Participant 1*

*“I was happy to spend the baseline and follow-up daytime. Very enjoyable.”*

*Participant 2*

*“Completing the daily activity diary was no problem. It was also good to keep a record so that I could go over past events.”*

*Participant 3*

*“The questions were relevant to the different factors affecting the ability to carry out the exercise. Pain, lifestyle and time can all be factors in the willingness to do differing amounts of exercise on any given day.”*

*Participant 4*

*“(5 weeks) Perfectly acceptable. The aim of the study is to improve the patient’s ability to move better and feel less discomfort. 5 weeks is a small amount of time to commit, compared to the quality of life that I feel I have regained.”*

*Participant 5*

#### Overall experience

Participants also had an opportunity to share any further thoughts, challenges, or positive experiences that may have occurred during their purposeful outdoor walks or throughout the study but have not been discussed so far in the interview. An interesting point was raised in relation to the wearability of the FC4 and Participant1 expressed he would wear the activity monitor only when had the intention to go for his outdoor walk. Others felt the study created a positive and beneficial habit to their daily routine and gained better self-confidence. Quotes from participants concerning their experience are outlined below.

*“I enjoyed looking at the map when I got back seeing the distance we had covered, where we had been and the time it had taken. I would put the monitor on and have it on my right hand - I would notice it had switched off, this puzzled me for a few days and was frustrating - that I knew I had done the mileage but it had not registered as it had turned off. I worked out if I flexed my wrist the back of my hand would sometimes turn the monitor off. So I started to wear it on my left hand & that’s my watch hand, so felt a bit foolish wearing (what appeared to be two watches ) I like to wear my watch & as I had difficulty reading the screen of the monitor (in the sunshine) did not want to not wear my watch.”*

*Participant 1*

*“I think this was a fantastic experience for me. It made me realise that I can do far more than I had thought I could, and with every week I felt stronger, faster, fitter, more stable and much more confident. When I did the baseline questions I thought I felt pretty confident, but I now realise I wasn’t anywhere near as confident as I needed to be.”*

*Participant 2*

*Having the activity monitor definitely made this study very enjoyable. I was determined to recover as quickly as possible from my hip surgery and completing this study gave me that extra determination. I will definitely make daily outdoor walking part of my daily life. I have walked kilometres that I never did before and I will definitely continue to exercise on a daily basis and hopefully in the near future I will be able to get back on my bike and include this as part of my daily fitness activity.*

*Participant 3*

*“Well my operated leg has definitely improved a lot, which I found out on this past Saturday my colleague got diagnosed with covid so I had to go into work early doors , I cant drive the van at the moment so I had to push 1200 L bins to the compactor area from all over the sight to empty them it involves most having to go through the link tunnel and that means pushing them uphill on part of the journey, my hips both hurt but next day it was only my right hip that’s giving me any pain. I know if this had happened a month ago I would have been sunk, so it has been a good thing for me.”*

*Participant 4*

*“It made me more communicative again, by passing the time of day with other walkers, particularly dog walkers as I love dogs, and cannot resist patting a friendly canine. Many happy little chats to brighten the day took place on my walks, hence my GPS shows several pauses on various days. It certainly brightened my mood.”*

*Participant 5*

## Discussion

This study was a small feasibility trial, to inform a follow up randomised pilot trial, with a convenience sample, that aimed to evaluate the appropriateness of outcome measures, recruitment, and adherence to the purposeful outdoor walking intervention monitored using a commercial activity monitor device to decide whether an effectiveness trial is warranted.

Five adults who had had THR surgery at least 3 months and at most 6 months ago, due to symptomatic hip osteoarthritis were recruited from the local community. Given the timeline and inclusion/exclusion criteria, the recruitment took almost 2 months. It is suggested that 1 or 2 participants per week are an acceptable recruitment rate for a clinical trial.^49^ Thus, the rate of our recruitment was below the average. Therefore, it should be emphasised that for a study of 12 participants with inclusion criteria, such as the one outlined here, recruitment may take up to 4 months to complete. It is recommended that 12 participants allows a clinical trial to provide a reliable answer to the question addressed.^50^

Current studies have provided evidence to support the use of wearable activity monitors in maintaining good health in older adults.^18,19^ Our findings also showed that the 5 weeks of outdoor walking intervention was accepted by the participant and full adherence was achieved.

Additionally, there was large variability between the weekly purposeful distance walked by the participants. The minimum purposeful walking distance increase was 69% and the maximum was 191% from the individuals' baseline distance. A minimum of 4.7 km per day was achieved by all participants.

Given there is currently no data available on the average outdoor distance walked for a healthy elderly adult or people post THR surgery, we compared our findings to studies by Schimpl et al.,^51^ Tang et al.,^8^ and Althoff et al.^52^ It is important to acknowledge that these studies are not restricted to outdoor walks only and did not utilise GPS to measure the daily distance walked. Schimpl et al.^51^ reported that an average healthy adult over the age of 60 years old, walks a mean of 5.5 km per day. However, the study by Althoff et al.^52^ which consisted of 68 million days of physical activity for 717,527 people, in 111 countries across the globe suggests that female adults over the age of 60 only achieve a daily distance of approximately 2.61 km per day and male achieve around 3.63 km per day. This distance was calculated using an arbitrary estimation (distance = step length x step count) data from an earlier study on the average step length of healthy adults and a group of THR patients. The estimation calculation is based on converting the 3600 steps for females and 5000 daily steps for males reported by Althoff et al.^52^ et al. by average step length of 0.725 m for adults over an age of 60 years old. Similarly, Tang et al.^8^ reported that at 3 months post THR, individuals were walking approximately 3 km (4526 steps multiplying by 0.652 m step length). Overall, all our study participants achieved beyond the estimated daily distance reported for healthy elderly adults. Further investigation on the data may also provide a platform to compare our findings to previous research which suggests that the risk of mortality is reduced with 7000 steps or more per day.^53^ Similarly, an arbitrary calculation converts this number of steps to a distance estimation of 5 km per day. At baseline only 2 participants (Participants IDs 1, and (2) were achieving this target, however, final week results showed that all participants were able to achieve this target on solely purposeful outdoor activity without taking any other indoor walking activity into account. Therefore, it may be concluded that increasing individuals’ baseline walking distance amount by 10% is feasible and beneficial to individuals, however, its efficacy should be assessed in a follow-up pilot study with a larger sample size compared against a control group.

Furthermore, evidence showed that there was a wide spread of distance that was achieved by individuals depending on their baseline abilities. Therefore, it is worth acknowledging that for any future pilot efficacy study with similar sample size, it is likely that we will again find very high variability in daily purposeful walk results. Thus, we should be prepared to carry out statistical analysis based on individual changes, to estimate any future effect size for a clinical trial.

When it comes to THR studies,^20,21^ the focus has been merely on the step count parameter and has not addressed the main gait adaptations e.g. shortened stride length,^7,22,23^ which persist even at 1-year after surgery. Bhave et al.^35^ found that gait analysis is valuable in identifying problems before and after THR. The visual, accurate, and reliable data obtained by gait analysis technology provide important objective data on individual walking patterns and modalities. Given the study design and its small sample size, we cannot statistically comment on the significant effect of the purposeful outdoor walking intervention on the gait parameters. However, our findings provide support to viability of a purposeful walk given there are improvements seen across almost all gait parameters (step length (operated side), walking speed, and cadence) in all participants. Therefore, the gait analysis using the GRAIL system was an appropriate test for such a study.

As stated earlier, a purposeful walk was the term that we used to refer to the outdoor walk that is recorded with a GPS sensor using the FC4 activity monitor. Therefore, compliance with the use of GPS was essential. All participants of our study admitted that FC4 encouraged them to go further and do a long daily walk. They also enjoyed looking at the map of the route they have walked daily and preferred it to simply seeing a daily step count. This finding is also in line with a recent survey on the perception of wearable technologies, which concluded that one of the new technologies that the majority of THR patients are willing to utilise in daily routine activities is the use of GPS.^54^

Another aim of this study was to help determine the feasibility of the outcome measures best suited to both participants and the objectives of this study. Hence, we selected a series of PROMS that were validated questionnaires and were previously utilised in THR studies. We considered only the MCID for the HOOS to be appropriate and findings showed that the average difference in HOOS outcome measures in the intervention group was 8.6 ± 7.2, which was below the reference for MCID suggesting a lack of clinically relevant meaningful difference from pre to post intervention. All three PROMs questionnaires provided different information. However, given the timeline post-surgery, and age (mostly retired), HOOS provided a more comprehensive set of detailed health outcome measures. HOOS has sections on physical activity level as well as specific questions on walking and therefore, it provides equivalent insight to mGES and PASE questions. Therefore, even though, all participants express their happiness about the time spent during the testing sessions, it was determined to use only the HOOS questionnaire in any upcoming efficacy studies.

Regarding the activity diary outcome measure, findings suggested that the participants found the questions straightforward and were able to answer them with ease and instinctively. However, completion of activity diary was a challenge for one participant (Participant 1). This was not due to the layout or type of questions, but mainly due to this individual’s reluctance to complete a writing task. One other person (Participant (2) also found scales difficult to complete and further explained that this was because the intensity of a walk and his fatigue changed over the course of the walk and it was hard to judge an average. Furthermore, we did not comment on the intensity of the daily walks measured with the Borg scale as all participants felt their outdoor walk never passed beyond moderate activity level, regardless of their daily targets. All feedback regarding the activity diary will be considered for any future design of a daily diary to ensure it is easier to complete.

The limitations in this study are mainly inherent to the study methodology. There was no formal power calculation and therefore the sample size was too small for statistical analysis or inadequate to reach a saturation in qualitative analysis. Moreover, the participants recruited in our study had their THR completed by different surgeons using different techniques and surgical approaches, which may influence their early post-operative recovery time.^55^ Furthermore, the exclusion criteria included other comorbidities and joint replacement, putting the sample at the risk of being homogenous. Outlined methodological limitation, in particular, small sample size, were adhered due to the timing of the study post COVID. Furthermore, we exclusion criteria such as, systematic disease effecting walking activity such as COPD, so we can reduce the chance of individuals being at risk while performing an outdoor walk at the timeline post THR. Furthermore, all included participants who were at least 3 months post-operation and could confirm they are discharged from their surgical care. Additionally, studies suggest that regardless of surgical approach or technique, at 3 months post-THR surgery, patients are ready to return to their normal activity.^56^ Importantly, in the absence of COMET guidelines on relevant outcome measure for evaluating individuals after THR surgery, we selected various outcome measured informed by previous publication and national reporting. A future patient and public involvement study is planned to include THR population in submission of an outcome measure best suited to assessment of THR population undergoing digital health related interventions. Lastly, there is limited evidence to support the accuracy and precision of the FC4 for monitoring individuals post THR. However, the intervention was designed based on individual walking amount in the first week and then a subsequent target was calculated for the individual wearing the same FC4 activity monitor. This approach was undertaken to limit the risk of the FC4 inaccuracy effecting the outcome of individuals daily walking amount.

## Conclusion

The objective of this small feasibility trial was to test the feasibility of the study methods and intervention delivery as well as the adherence to the personalised outdoor purposeful walking intervention in preparation for future trials. Although the PROMS selected were all relevant to this cohort, future research will only include the HOOS questionnaire, as it provides the most comprehensive and relevant set of subjective outcomes. Gait analysis was well received by all participants and the gait parameters selected provided great insight into the effects of the intervention on walking recovery post-THR surgery. Furthermore, the purposeful walking intervention was acceptable to all participants and should be considered without being amended in any future efficacy trials. Lastly, it is important to note that there was a wide spread of distance that was achieved by individuals and therefore a future trial with a similar sample size and variability in data should consider statistical analysis based on individual changes, in order to estimate an effect size for a clinical trial.

## Supplemental Material

Supplemental Material - A feasibility study to evaluate a purposeful walk intervention with a distance goal using a commercially available activity monitor in elderly people post total hip replacement surgery Click here for additional data file. Supplemental Material for A feasibility study to evaluate a purposeful walk intervention with a distance goal using a commercially available activity monitor in elderly people post total hip replacement surgery by Shayan Bahadori, Jonathan Mark Williams, Sarah Collard and Ian Swain in Journal of Rehabilitation and Assistive Technologies Engineering

Supplemental Material - A feasibility study to evaluate a purposeful walk intervention with a distance goal using a commercially available activity monitor in elderly people post total hip replacement surgery Click here for additional data file. Supplemental Material for A feasibility study to evaluate a purposeful walk intervention with a distance goal using a commercially available activity monitor in elderly people post total hip replacement surgery by Shayan Bahadori, Jonathan Mark Williams, Sarah Collard and Ian Swain in Journal of Rehabilitation and Assistive Technologies Engineering

Supplemental Material - A feasibility study to evaluate a purposeful walk intervention with a distance goal using a commercially available activity monitor in elderly people post total hip replacement surgery Click here for additional data file. Supplemental Material for A feasibility study to evaluate a purposeful walk intervention with a distance goal using a commercially available activity monitor in elderly people post total hip replacement surgery by Shayan Bahadori, Jonathan Mark Williams, Sarah Collard and Ian Swain in Journal of Rehabilitation and Assistive Technologies Engineering

Supplemental Material - A feasibility study to evaluate a purposeful walk intervention with a distance goal using a commercially available activity monitor in elderly people post total hip replacement surgery Click here for additional data file. Supplemental Material for A feasibility study to evaluate a purposeful walk intervention with a distance goal using a commercially available activity monitor in elderly people post total hip replacement surgery by Shayan Bahadori, Jonathan Mark Williams, Sarah Collard and Ian Swain in Journal of Rehabilitation and Assistive Technologies Engineering

## References

[1] Culliford D Maskell J Judge A , et al. Future projections of total hip and knee arthroplasty in the UK: results from the UK clinical practice research Datalink. Osteoarthritis Cartilage 2015; 23(4): 594–600. DOI: 10.1016/j.joca.2014.12.022 25579802

[2] National Joint Registry, N . National joint registry 19th annual report. London, UK: National Joint Registry, N, 2022. 36516281

[3] Chen A Gupte C Akhtar K , et al. The global economic cost of osteoarthritis: how the UK compares. Arthritis 2012; 6: 698709. DOI: 10.1155/2012/698709 PMC3467755 23082249

[4] Astephen Wilson JL Lamontagne M Wilson DR , et al. Patient-specific functional analysis: the key to the next revolution towards the treatment of hip and knee osteoarthritis. J Orthop Res 2019; 37(8): 1754–1759. DOI: 10.1002/jor.24317 31042316

[5] Beswick AD Wylde V Gooberman-Hill R , et al. What proportion of patients report long-term pain after total hip or knee replacement for osteoarthritis? A systematic review of prospective studies in unselected patients. BMJ Open 2012; 2(1): e000435. DOI: 10.1136/bmjopen-2011-000435 PMC3289991 22357571

[6] Smith TO Mansfield M Dainty J , et al. Does physical activity change following hip and knee replacement? Matched case-control study evaluating physical activity scale for the elderly data from the osteoarthritis initiative. Physiotherapy 2018; 104(1): 80–90. DOI: 10.1016/j.physio.2017.02.001 28917522

[7] Beaulieu ML Lamontagne M Beaulé PE . Lower limb biomechanics during gait do not return to normal following total hip arthroplasty. Gait Posture 2010; 32(2): 269–273. DOI: 10.1016/j.gaitpost.2010.05.007. 20541940

[8] Tang A Behery OA Singh V , et al. Do physical activity and sleep correlate with patient-reported outcomes in total hip arthroplasty? The Journal of Hip Surgery 2021; 05(02): 47–54.

[9] Crizer MP Kazarian GS Fleischman AN , et al. Stepping toward objective outcomes: a prospective analysis of step count after total joint arthroplasty. J Arthroplasty 2017; 32(9s): S162–s165. DOI: 10.1016/j.arth.2017.02.058 28343831

[10] Holl S Blum A Gosheger G , et al. Clinical outcome and physical activity measured with StepWatch 3 Activity monitor after minimally invasive total hip arthroplasty. J Orthop Surg Res 2018; 13(1): 148. DOI: 10.1186/s13018-018-0775-4 29907134 PMC6003151

[11] Withers TM Lister S Sackley C , et al. Is there a difference in physical activity levels in patients before and up to one year after unilateral total hip replacement? A systematic review and meta-analysis. Clin Rehabil 2017; 31(5): 639–650. 27773874 10.1177/0269215516673884 PMC5407512

[12] Harding P Holland AE Delany C , et al. Do activity levels increase after total hip and knee arthroplasty? Clin Orthop Relat Res 2014; 472(5): 1502–1511. DOI: 10.1007/s11999-013-3427-3 24353051 PMC3971219

[13] Bahadori S Collard S Williams JM , et al. Why do people undergo THR and what do they expect to gain—a comparison of the views of patients and health care professionals. Journal of Patient Experience 2020; 7(6): 1778–1787. 33457643 10.1177/2374373520956735 PMC7786753

[14] Salpakoski A Törmäkangas T Edgren J , et al. Walking recovery after a hip fracture: a prospective follow-up study among community-dwelling over 60-year old men and women. Biomed Res Int 2014; 2014: 289549. DOI: 10.1155/2014/289549. 24511530 PMC3912885

[15] Simonsick EM Guralnik JM Volpato S , et al. Just get out the door! Importance of walking outside the home for maintaining mobility: findings from the women’s health and aging study. J Am Geriatr Soc 2005; 53(2): 198–203. 15673341 10.1111/j.1532-5415.2005.53103.x

[16] Henriksen A Haugen Mikalsen M Woldaregay AZ , et al. Using fitness trackers and smartwatches to measure physical activity in research: analysis of consumer wrist-worn wearables. J Med Internet Res 2018; 20(3): e110. DOI: 10.2196/jmir.9157 29567635 PMC5887043

[17] Bahadori S . Which commercial activity monitor for the THR population? An evaluation of commercial activity monitors to measure walking distance, 2021.

[18] Gartner . Connected health information in Canada a benefits evaluation study. Chandigarh: CH Infoway, 2018. file:///C:/Users/sbahadori/Downloads/connected_health_EN.pdf

[19] Kononova A Li L Kamp K , et al. The use of wearable activity trackers among older adults: focus group study of tracker perceptions, motivators, and barriers in the maintenance stage of behavior change. JMIR Mhealth Uhealth 2019; 7(4): e9832. 30950807 10.2196/mhealth.9832 PMC6473213

[20] Toogood P Abdel M Spear J , et al. The monitoring of activity at home after total hip arthroplasty. Bone Joint Lett J 2016; 98-B(11): 1450–1454. DOI: 10.1302/0301-620x.98b11.bjj-2016-0194.r1 27803219

[21] Van der Walt N Salmon LJ Gooden B , et al. Feedback from activity trackers improves daily step count after knee and hip arthroplasty: a randomized controlled trial. J Arthroplasty 2018; 33(11): 3422–3428. DOI: 10.1016/j.arth.2018.06.024 30017217

[22] Bahadori S Spatio-temporal characteristics of total hip replacement patients and their relevance to walking, 2020. *(Unpublished Work)* .

[23] Ewen AM Stewart S St Clair Gibson A , et al. Post-operative gait analysis in total hip replacement patients-a review of current literature and meta-analysis. Gait Posture 2012; 36(1): 1–6. DOI: 10.1016/j.gaitpost.2011.12.024 22410129

[24] Morris CE Garner JC Owens SG , et al. A prospective study comparing distance-based vs. time-based exercise prescriptions of walking and running in previously sedentary overweight adults. Int J Exerc Sci 2017; 10(5): 782. 28966715 10.70252/YJIX2289 PMC5609661

[25] Camarri B Eastwood PR Cecins NM , et al. Six minute walk distance in healthy subjects aged 55–75 years. Respir Med 2006; 100(4): 658–665. 16229997 10.1016/j.rmed.2005.08.003

[26] Troosters T Gosselink R Decramer M . Six minute walking distance in healthy elderly subjects. Eur Respir J 1999; 14(2): 270–274. 10515400 10.1034/j.1399-3003.1999.14b06.x

[27] Brown M Hislop HJ Waters RL , et al. Walking efficiency before and after total hip replacement. Phys Ther 1980; 60(10): 1259–1263. DOI: 10.1093/ptj/60.10.1259 7443787

[28] Lancaster GA Thabane L . Guidelines for reporting non-randomised pilot and feasibility studies. BioMed Central 2019; 5: 1–6. 10.1186/s40814-019-0499-1 PMC6778655 31608150

[29] Von Elm E Altman DG Egger M , et al. The strengthening the reporting of observational studies in epidemiology (STROBE) statement: guidelines for reporting observational studies. Ann Intern Med 2007; 147(8): 573–577. 17938396 10.7326/0003-4819-147-8-200710160-00010

[30] Sloot LH van der Krogt MM Harlaar J . Self-paced versus fixed speed treadmill walking. Gait Posture 2014; 39(1): 478–484. DOI: 10.1016/j.gaitpost.2013.08.022. 24055003

[31] Bahadori S Wainwright TW . Lower limb biomechanical analysis of healthy participants. J Vis Exp 2020;(158): e60720. 10.3791/60720 32364546

[32] van den Bogert AJ Geijtenbeek T Even-Zohar O , et al. A real-time system for biomechanical analysis of human movement and muscle function [journal article]. Med Biol Eng Comput 2013; 51(10): 1069–1077. DOI: 10.1007/s11517-013-1076-z 23884905 PMC3751375

[33] Bahadori S Immins T Wainwright TW . Reliability of gait parameters in male and female healthy adults during self-paced treadmill-based walking. Int J Ther Rehabil 2020; 27(9): 1–18.

[34] Kribus-Shmiel L Zeilig G Sokolovski B , et al. How many strides are required for a reliable estimation of temporal gait parameters? Implementation of a new algorithm on the phase coordination index. PLoS One 2018; 13(2): e0192049–e0192049. DOI: 10.1371/journal.pone.0192049 29420580 PMC5805232

[35] Bhave A Marker DR Seyler TM , et al. Functional problems and treatment solutions after total hip arthroplasty. J Arthroplasty 2007; 22(6): 116–124. 10.1016/j.arth.2007.04.025 17823029

[36] Nilsdotter AK Lohmander LS Klassbo M , et al. Hip disability and osteoarthritis outcome score (HOOS)--validity and responsiveness in total hip replacement. BMC Musculoskelet Disord 2003; 4: 10. DOI: 10.1186/1471-2474-4-10 12777182 PMC161815

[37] Goodman SM Mehta BY Mandl LA , et al. Validation of the hip disability and osteoarthritis outcome score and knee injury and osteoarthritis outcome score pain and function subscales for use in total hip replacement and total knee replacement Clinical Trials. J Arthroplasty 2020; 35(5): 1200–1207.e1204. DOI: 10.1016/j.arth.2019.12.038 31952945 PMC7193650

[38] Soh S-E Harris IA Cashman K , et al. Minimal clinically important changes in HOOS-12 and KOOS-12 scores following joint replacement. JBJS 2022; 104(11): 980–987. DOI: 10.2106/jbjs.21.00741 35648064

[39] Washburn RA Smith KW Jette AM , et al. The physical activity scale for the elderly (PASE): development and evaluation. J Clin Epidemiol 1993; 46(2): 153–162. DOI: 10.1016/0895-4356(93)90053-4. 8437031

[40] Svege I Kolle E Risberg MA . Reliability and validity of the physical activity scale for the elderly (PASE) in patients with hip osteoarthritis. BMC Musculoskelet Disord 2012; 13: 26. DOI: 10.1186/1471-2474-13-26 22353558 PMC3305439

[41] Newell AM VanSwearingen JM Hile E , et al. The modified gait efficacy scale: establishing the psychometric properties in older adults. Phys Ther 2012; 92(2): 318–328. 22074940 10.2522/ptj.20110053 PMC3269773

[42] Weijer R Hoozemans M van Dieën J , et al. Construct validity and reliability of the modified gait efficacy scale for older adults. Disabil Rehabil 2020; 44(11): 2464–2469. 33174486 10.1080/09638288.2020.1840638

[43] Fransen BL Pijnappels M Butter IK , et al. Patients’ perceived walking abilities, daily-life gait behavior and gait quality before and 3 months after total knee arthroplasty. Arch Orthop Trauma Surg 2021; 142(6): 1189–1196. DOI: 10.1007/s00402-021-03915-y 33956227 PMC9110478

[44] Xia A Zhang X Liu Y , et al. Validation of the Chinese version of the modified gait efficacy scale for patients removing ilizarov external fixation device for over one year. Patient Prefer Adherence 2020; 14: 1307. 32801661 10.2147/PPA.S250440 PMC7398744

[45] Oosting E Jans MP Dronkers JJ , et al. Preoperative home-based physical therapy versus usual care to improve functional health of frail older adults scheduled for elective total hip arthroplasty: a pilot randomized controlled trial. Arch Phys Med Rehabil 2012; 93(4): 610–616. 22365481 10.1016/j.apmr.2011.11.006

[46] DeJonckheere M Vaughn LM Semistructured interviewing in primary care research: a balance of relationship and rigour. Fam Med Community Health 2019; 7(2). 10.1136/fmch-2018-000057 PMC6910737 32148704

[47] Braun V Clarke V . Using thematic analysis in psychology. Qual Res Psychol 2006; 3(2): 77–101. DOI: 10.1191/1478088706qp063oa

[48] Vaismoradi M Jones J Turunen H , et al. Theme development in qualitative content analysis and thematic analysis. J Nurs Educ Pract 2016; 6(5).

[49] Walters SJ Bonacho dos Anjos Henriques-Cadby I Bortolami O , et al. Recruitment and retention of participants in randomised controlled trials: a review of trials funded and published by the United Kingdom health technology assessment programme. BMJ Open 2017; 7(3): e015276. DOI: 10.1136/bmjopen-2016-015276 PMC5372123 28320800

[50] Julious S . Sample size of 12 per group rule of thumb for a pilot study. Pharmaceut Stat 2005; 4(4): 287–291.

[51] Schimpl M Moore C Lederer C , et al. Association between walking speed and age in healthy, free-living individuals using mobile accelerometry—a cross-sectional study. PLoS One 2011; 6(8): e23299. 21853107 10.1371/journal.pone.0023299 PMC3154324

[52] Althoff T Sosič JL Hicks AC , et al. Large-scale physical activity data reveal worldwide activity inequality. Nature 2017; 547(7663): 336–339. 28693034 10.1038/nature23018 PMC5774986

[53] Paluch AE Gabriel KP Fulton JE , et al. Steps per day and all-cause mortality in middle-aged adults in the coronary artery risk development in young adults study. JAMA Netw Open 2021; 4(9): e2124516–e2124516. DOI: 10.1001/jamanetworkopen.2021.24516 34477847 PMC8417757

[54] Kurtz SM Higgs GB Chen Z , et al. Patient perceptions of wearable and smartphone technologies for remote outcome monitoring in patients who have hip osteoarthritis or arthroplasties. J Arthroplasty 2022; 37(7S): S488–S492.e2. 35277311 10.1016/j.arth.2022.02.026

[55] Aggarwal V Iorio R Zuckerman J , et al. Surgical approaches for primary total hip arthroplasty from charnley to now. JBJS Reviews 2020; 8: e0058. 32105236 10.2106/JBJS.RVW.19.00058

[56] Jones DL Westby MD Greidanus N , et al. Update on hip and knee arthroplasty: current state of evidence. Arthritis Rheum 2005; 53(5): 772–780. 16208670 10.1002/art.21465

