# Supplementary material for: A feasibility study to evaluate a purposeful walk intervention with a distance goal using a commercially available activity monitor in elderly people post total hip replacement surgery
Source: J Rehabil Assist Technol Eng. 2023 Aug 22;10:20556683231195927. doi: 10.1177/20556683231195927 (PMC10447967; doi:10.1177/20556683231195927)
Supplement: Supplemental Material - A feasibility study to evaluate a purposeful walk intervention with a distance goal using a commercially available activity monitor in elderly people post total hip replacement surgery [file sj-pdf-2-jrt-10.1177_20556683231195927.pdf]

PASE
Physical Activity Scale for the Elderly

New England Research Institutes

© 1991
New England Research Institutes, Inc.
9 Galen Street   Watertown, MA   02472
617.923.7747   http://www.neriscience.com

INSTRUCTIONS:

Please complete this questionnaire by either circling the correct response or filling in the blank. Here is an example:

During the past 7 days, how often have you seen the sun?

[0.] NEVER	[1.] SELDOM (1-2 DAYS)	[2.] SOMETIMES (3-4 DAYS)	[3.] OFTEN (5-7 DAYS)

Answer all items as accurately as possible. All information is strictly confidential.

LEISURE TIME ACTIVITY

1. Over the past 7 days, how often did you participate in sitting activities such as reading, watching TV or doing handcrafts?

[0.] NEVER ↓ GO TO Q.#2 | [1.] SELDOM (1-2 DAYS) ↓ | [2.] SOMETIMES (3-4 DAYS) ↓ | [3.] OFTEN (5-7 DAYS) ↓

1a. What were these activities?

____

1b. On average, how many hours per day did you engage in these sitting activities?

[1.] LESS THAN 1 HOUR [2.] 1 BUT LESS THAN 2 HOURS

[3.] 2-4 HOURS [4.] MORE THAN 4 HOURS

2. Over the past 7 days, how often did you take a walk outside your home or yard for any reason? For example, for fun or exercise, walking to work, walking the dog, etc.?

[0.] NEVER ↓ GO TO Q.#3 | [1.] SELDOM (1-2 DAYS) ↓ | [2.] SOMETIMES (3-4 DAYS) ↓ | [3.] OFTEN (5-7 DAYS) ↓

2a. On average, how many hours per day did you spend walking?

[1.] LESS THAN 1 HOUR [2.] 1 BUT LESS THAN 2 HOURS

[3.] 2-4 HOURS [4.] MORE THAN 4 HOURS

3. Over the past 7 days, how often did you engage in light sport or recreational activities such as bowling, golf with a cart, shuffleboard, fishing from a boat or pier or other similar activities?

[0.] NEVER ↓ GO TO Q.#4 | [1.] SELDOM (1-2 DAYS) ↓ | [2.] SOMETIMES (3-4 DAYS) ↓ | [3.] OFTEN (5-7 DAYS) ↓

3a. What were these activities?

____

3b. On average, how many hours per day did you engage in these light sport or recreational activities?

[1.] LESS THAN 1 HOUR [2.] 1 BUT LESS THAN 2 HOURS

[3.] 2-4 HOURS [4.] MORE THAN 4 HOURS

4. Over the past 7 days, how often did you engage in moderate sport and recreational activities such as doubles tennis, ballroom dancing, hunting, ice skating, golf without a cart, softball or other similar activities?

[0.] NEVER ↓ GO TO Q.#5 | [1.] SELDOM (1-2 DAYS) ↓ | [2.] SOMETIMES (3-4 DAYS) ↓ | [3.] OFTEN (5-7 DAYS) ↓

4a. What were these activities?

____

4b. On average, how many hours per day did you engage in these moderate sport and recreational activities?

[1.] LESS THAN 1 HOUR [2.] 1 BUT LESS THAN 2 HOURS

[3.] 2-4 HOURS [4.] MORE THAN 4 HOURS

5. Over the past 7 days, how often did you engage in strenuous sport and recreational activities such as jogging, swimming, cycling, singles tennis, aerobic dance, skiing (downhill or cross-country) or other similar activities?

[0.] NEVER ↓ GO TO Q.#6

[1.] SELDOM (1-2 DAYS) ↓

[2.] SOMETIMES (3-4 DAYS) ↓

[3.] OFTEN (5-7 DAYS) ↓

5a. What were these activities?

____

5b. On average, how many hours per day did you engage in these strenuous sport and recreational activities?

[1.] LESS THAN 1 HOUR [2.] 1 BUT LESS THAN 2 HOURS

[3.] 2-4 HOURS [4.] MORE THAN 4 HOURS

6. Over the past 7 days, how often did you do any exercises specifically to increase muscle strength and endurance, such as lifting weights or pushups, etc.?

[0.] NEVER ↓ GO TO Q.#7

[1.] SELDOM (1-2 DAYS) ↓

[2.] SOMETIMES (3-4 DAYS) ↓

[3.] OFTEN (5-7 DAYS) ↓

6a. What were these activities?

____

6b. On average, how many hours per day did you engage in exercises to increase muscle strength and endurance?

[1.] LESS THAN 1 HOUR [2.] 1 BUT LESS THAN 2 HOURS

[3.] 2-4 HOURS [4.] MORE THAN 4 HOURS

HOUSEHOLD ACTIVITY

7. During the past 7 days, have you done any light housework, such as dusting or washing dishes?

   [1.] NO [2.] YES

8. During the past 7 days, have you done any heavy housework or chores, such as vacuuming, scrubbing floors, washing windows, or carrying wood?

   [1.] NO [2.] YES

9. During the past 7 days, did you engage in any of the following activities?

   Please answer YES or NO for each item.

		NO	YES
a.	Home repairs like painting, wallpapering, electrical work, etc.	1	2
b.	Lawn work or yard care, including snow or leaf removal, wood chopping, etc.	1	2
c.	Outdoor gardening	1	2
d.	Caring for an other person, such as children, dependent spouse, or an other adult	1	2

WORK-RELATED ACTIVITY

10. During the past 7 days, did you work for pay or as a volunteer?

[1.] NO [2.] YES

10a. How many hours per week did you work for pay and/or as a volunteer?

_______________ HOURS

10b. Which of the following categories best describes the amount of physical activity required on your job and/or volunteer work?

[1] Mainly sitting with slight arm movements.
[**Examples:** office worker, watchmaker, seated assembly line worker, bus driver, etc.]

[2] Sitting or standing with some walking.
[**Examples:** cashier, general office worker, light tool and machinery worker.]

[3] Walking, with some handling of materials generally weighing less than 50 pounds.
[**Examples:** mailman, waiter/waitress, construction worker, heavy tool and machinery worker.]

[4] Walking and heavy manual work often requiring handling of materials weighing over 50 pounds.
[**Examples:** lumberjack, stone mason, farm or general laborer.]

THANK YOU FOR TAKING THE TIME AND EFFORT

TO COMPLETE THIS QUESTIONNAIRE!
