# Supplementary material for: A feasibility study to evaluate a purposeful walk intervention with a distance goal using a commercially available activity monitor in elderly people post total hip replacement surgery
Source: J Rehabil Assist Technol Eng. 2023 Aug 22;10:20556683231195927. doi: 10.1177/20556683231195927 (PMC10447967; doi:10.1177/20556683231195927)
Supplement: Supplemental Material - A feasibility study to evaluate a purposeful walk intervention with a distance goal using a commercially available activity monitor in elderly people post total hip replacement surgery [file sj-pdf-3-jrt-10.1177_20556683231195927.pdf]

Appendix.

The Modified Gait Efficacy Scale (mGES)

1. How much confidence do you have that you would be able to safely walk on a level surface such as a hardwood floor?

1	2	3	4	5	6	7	8	9	10

No Confidence   Complete Confidence

2. How much confidence do you have that you would be able to safely walk on grass?

1	2	3	4	5	6	7	8	9	10

No Confidence   Complete Confidence

3. How much confidence do you have that you would be able to safely walk over an obstacle in your path?

1	2	3	4	5	6	7	8	9	10

No Confidence   Complete Confidence

4. How much confidence do you have that you would be able to safely step down from a curb?

1	2	3	4	5	6	7	8	9	10

No Confidence   Complete Confidence

5. How much confidence do you have that you would be able to safely step up onto a curb?

1	2	3	4	5	6	7	8	9	10

No Confidence   Complete Confidence

6. How much confidence do you have that you would be able to safely walk up stairs if you are holding on to a railing?

1	2	3	4	5	6	7	8	9	10

No Confidence   Complete Confidence

7. How much confidence do you have that you would be able to safely walk down stairs if you are holding on to a railing?

1	2	3	4	5	6	7	8	9	10

No Confidence   Complete Confidence

8. How much confidence do you have that you would be able to safely walk up stairs if you are NOT holding on to a railing?

1	2	3	4	5	6	7	8	9	10

No Confidence   Complete Confidence

(Continued)

Downloaded from https://academic.oup.com/ptj/article/92/2/318/2703187 by Bournemouth University user on 14 September 2021

Appendix.
Continued

9. How much confidence do you have that you would be able to safely walk down stairs if you are NOT holding on to a railing?

1	2	3	4	5	6	7	8	9	10

No Confidence Complete Confidence

10. How much confidence do you have that you would be able to safely walk a long distance such as ½ mile?

1	2	3	4	5	6	7	8	9	10

No Confidence Complete Confidence

Downloaded from https://academic.oup.com/ptj/article/92/2/318/2703187 by Bournemouth University user on 14 September 2021
