# Supplementary material for: A feasibility study to evaluate a purposeful walk intervention with a distance goal using a commercially available activity monitor in elderly people post total hip replacement surgery
Source: J Rehabil Assist Technol Eng. 2023 Aug 22;10:20556683231195927. doi: 10.1177/20556683231195927 (PMC10447967; doi:10.1177/20556683231195927)
Supplement: Supplemental Material - A feasibility study to evaluate a purposeful walk intervention with a distance goal using a commercially available activity monitor in elderly people post total hip replacement surgery [file sj-pdf-4-jrt-10.1177_20556683231195927.pdf]

Appendix A - HOOS Questionnaire.
Appendix B - PASE Questionnaire.
Appendix C - mGES Questionnaire.

Appendix D - Activity Diary.

Activity diary

NAME……………………………………………………………

[illegible] 2. We would like you to record the amount of distance you walked in kilometres (Km) as recorded on your activity monitor and how intensely you felt you were exercising on average (Borg Scale). The Borg Scale is provided for you below, please use this as a guide and score yourself out of 10, (e.g. 1/10 representing hardly any exertion and 10/10 representing maximum effort).

Bring your activity diary with you to your follow-up assessment for review by the research team.

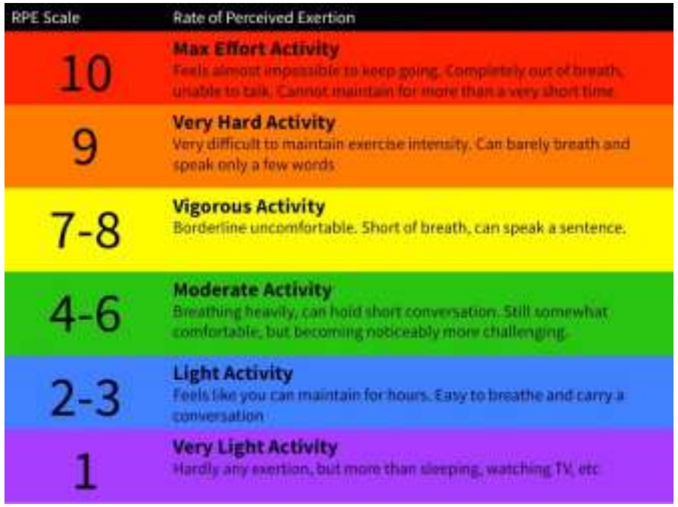

**Date** *(Please write the date for every entry below)*	***DO NOT WRITE ANYTHING HERE***	Daily Walk	Was your daily outdoor walk affected by any of the following? Please place an **X** on the scale to indicate your estimate level of fatigue, pain, and general busyness.	Please use this section to expand on any condition/feelings which may have affected your daily outdoor walk for this day
	Total Distance *(Km)*		**Fatigue**<br>Not at all — Very much so<br>**Pain**<br>Not at all — Very much so<br>**General Busyness**<br>Not at all — Very much so	
	Intensity *(Borg Scale i.e. 4/10)*			

	Total Distance *(km)*		**Fatigue**<br>Not at all — Very much so<br>**Pain**<br>Not at all — Very much so<br>**General Busyness**<br>Not at all — Very much so	

	Intensity *(Borg Scale i.e. 4/10)*			
	Total Distance *(Km)*<br><br>Intensity *(Borg Scale i.e. 4/10)*		**Fatigue**<br>Not at all Very much so<br>Not at all **Pain** Very much so<br>**General Busyness**<br>Not at all Very much so	
	Total Distance *(Km)*		**Fatigue**<br>Not at all Very much so<br>Not at all **Pain** Very much so<br>**General Busyness**<br>Not at all Very much so	

	Intensity *(Borg Scale i.e. 4/10)*			
	Total Distance *(Km)*		**Fatigue**<br>Not at all — Very much so	
	Intensity *(Borg Scale i.e. 4/10)*		**Pain**<br>Not at all — Very much so<br>**General Busyness**<br>Not at all — Very much so	

	Total Distance *(Km)*<br><br>Intensity *(Borg Scale i.e. 4/10)*		**Fatigue**<br>Not at all — Very much so<br>**Pain**<br>Not at all — Very much so<br>**General Busyness**<br>Not at all — Very much so	
	Total Distance *(km)*<br><br>Intensity *(Borg Scale i.e. 4/10)*		**Fatigue**<br>Not at all — Very much so<br>**Pain**<br>Not at all — Very much so<br>**General Busyness**<br>Not at all — Very much so	

	Total Distance *(Km)*		**Fatigue**<br>Not at all Very much so	
	Intensity *(Borg Scale i.e. 4/10)*		Not at all **Pain** Very much so<br>**General Busyness**<br>Not at all Very much so	
	Total Distance *(Km)*		**Fatigue**<br>Not at all Very much so	
	Intensity *(Borg Scale i.e. 4/10)*		Not at all **Pain** Very much so<br>**General Busyness**<br>Not at all Very much so	

	Total Distance *(Km)*<br><br>Intensity *(Borg Scale i.e. 4/10)*		**Fatigue**<br>Not at all Very much so<br>**Pain**<br>Not at all Very much so<br>**General Busyness**<br>Not at all Very much so	
	Total Distance *(Km)*<br><br>Intensity *(Borg Scale i.e. 4/10)*		**Fatigue**<br>Not at all Very much so<br>**Pain**<br>Not at all Very much so<br>**General Busyness**<br>Not at all Very much so	

	Total Distance *(km)*		**Fatigue**<br>Not at all — Very much so<br>**Pain**<br>Not at all — Very much so	
	Intensity *(Borg Scale i.e. 4/10)*		**General Busyness**<br>Not at all — Very much so	
	Total Distance *(Km)*		**Fatigue**<br>Not at all — Very much so<br>**Pain**<br>Not at all — Very much so	
	Intensity *(Borg Scale i.e. 4/10)*		**General Busyness**<br>Not at all — Very much so	

	Total Distance *(Km)*<br><br>Intensity *(Borg Scale i.e. 4/10)*		**Fatigue**<br>Not at all ... Very much so<br>**Pain**<br>Not at all ... Very much so<br>**General Busyness**<br>Not at all ... Very much so	
	Total Distance *(Km)*		**Fatigue**<br>Not at all ... Very much so<br>**Pain**<br>Not at all ... Very much so<br>**General Busyness**<br>Not at all ... Very much so	

	Intensity *(Borg Scale i.e. 4/10)*			
	Total Distance *(Km)*		**Fatigue**<br>Not at all Very much so<br>Not at all **Pain** Very much so<br>**General Busyness**<br>Not at all Very much so	
	Intensity *(Borg Scale i.e. 4/10)*			
	Total Distance *(km)*		**Fatigue**<br>Not at all Very much so<br>Not at all **Pain** Very much so<br>**General Busyness**<br>Not at all Very much so	

	Intensity *(Borg Scale i.e. 4/10)*			
	Total Distance *(Km)*		**Fatigue**<br>Not at all — Very much so<br>**Pain**<br>Not at all — Very much so<br>**General Busyness**<br>Not at all — Very much so	
	Intensity *(Borg Scale i.e. 4/10)*			

	Total Distance *(Km)*<br><br>Intensity *(Borg Scale i.e. 4/10)*		**Fatigue**<br>Not at all ——— Very much so<br>**Pain**<br>Not at all ——— Very much so<br>**General Busyness**<br>Not at all ——— Very much so	
	Total Distance *(km)*<br><br>Intensity *(Borg Scale i.e. 4/10)*		**Fatigue**<br>Not at all ——— Very much so<br>**Pain**<br>Not at all ——— Very much so<br>**General Busyness**<br>Not at all ——— Very much so	

	Total Distance *(Km)*		**Fatigue**<br>Not at all ——— Very much so<br>**Pain**<br>Not at all ——— Very much so<br>**General Busyness**<br>Not at all ——— Very much so	
	Intensity *(Borg Scale i.e. 4/10)*			
	Total Distance *(Km)*		**Fatigue**<br>Not at all ——— Very much so<br>**Pain**<br>Not at all ——— Very much so<br>**General Busyness**<br>Not at all ——— Very much so	
	Intensity *(Borg Scale i.e. 4/10)*			

	Total Distance *(Km)*<br><br>Intensity *(Borg Scale i.e. 4/10)*		**Fatigue**<br>Not at all — Very much so<br>**Pain**<br>Not at all — Very much so<br>**General Busyness**<br>Not at all — Very much so	
	Total Distance *(Km)*		**Fatigue**<br>Not at all — Very much so<br>**Pain**<br>Not at all — Very much so<br>**General Busyness**<br>Not at all — Very much so	

	Intensity *(Borg Scale i.e. 4/10)*			
	Total Distance *(km)*<br>Intensity *(Borg Scale i.e. 4/10)*		**Fatigue**<br>Not at all Very much so<br>**Pain**<br>Not at all Very much so<br>**General Busyness**<br>Not at all Very much so	
	Total Distance *(Km)*		**Fatigue**<br>Not at all Very much so<br>**Pain**<br>Not at all Very much so<br>**General Busyness**<br>Not at all Very much so	

	Intensity *(Borg Scale i.e. 4/10)*			
	Total Distance *(Km)*		**Fatigue**<br>Not at all Very much so<br>**Pain**<br>Not at all Very much so<br>**General Busyness**<br>Not at all Very much so	
	Intensity *(Borg Scale i.e. 4/10)*			

	Total Distance *(Km)*		**Fatigue**<br>Not at all — Very much so	
	Intensity *(Borg Scale i.e. 4/10)*		**Pain**<br>Not at all — Very much so<br>**General Busyness**<br>Not at all — Very much so	

Appendix E - Interview topic guide.

Subject 1: Activity Monitor

What was it like to wear the activity monitor?

What was it like to use the activity monitor?

Did the activity monitor encourage you to do a daily walk (achieved your daily goals)? (Remember to ask 'How' if they didn't expand on it)

What were your thoughts on the use of activity monitors prior to study?

Would you consider buying an activity monitor? Could you please explain why.

Subject 2: Purposeful walking:

How did you feel about going for your outdoor walks?

Did you find your goals manageable? Could you please explain

Did you find your daily distance goals helpful with increasing your daily activity? Could you please explain

Subject 3: Time (testing/intervention)

How did you feel about the amount of time you spent with us on baseline day and follow up day testing?

How did you feel about being part of a study that involves you for 5 weeks?

What did you think about the layout of the activity diary?

How did you feel about completing the daily activity diary?

What are your thoughts on the style of questions asked in the activity diary? Could you please explain

Subject 4: Explore

Were there any challenges that you would like to share in regard to doing your daily outdoor walking?

Were there any positive experiences that you would like to share in regard to doing your daily outdoor walking.
